# Conservation and coexistence at a crossroads

**DOI:** 10.1111/cobi.14433

**Published:** 2024-12-19

**Authors:** Simon Pooley

**Affiliations:** ^1^ School of Social Sciences Birkbeck University of London London UK

**Keywords:** complexity, conservation policy, Global Biodiversity Framework, human‐wildlifecoexistence, indicators andmetrics

Conservationists continue to wrestle with antithetical urges toward universality and diversity. A quest for blueprints for saving biodiversity exists sympatrically with acknowledgment of the plurality of ways of living in the natural world. Current attempts to integrate emergent thinking on human–wildlife coexistence into the requirements of the Kunming–Montreal Global Biodiversity Framework (GBF) bring this into sharp focus. This is a moment worthy of pause and consideration of the implications of integrating coexistence into existing conservation paradigms.

Conservation biology has evolved into conservation science, enlarging its remit to grapple with the undeniable ascendancy of humans in driving the planet out of the Holocene. Despite this, the field is still firmly rooted in the natural sciences, seeking better scientific descriptions of natural systems and species, sophisticated models, and general laws for social–ecological systems (so conceived) in quest of evidence‐based, generalizable approaches for averting biodiversity loss. These aspirations are consistent with those of international policy makers aiming to regulate humanity out of planetary disaster.

The GBF requires measurable steps toward solving specific biodiversity‐related challenges. Meeting the 23 targets for 2030 (notably those relating to tools and solutions) requires agreement on concepts, indicators, standards, and best processes and practices (Convention on Biological Diversity [CBD], 2024) to facilitate uniform assessments across all 168 signatory countries.

Yet, even social scientists adopting generalizable social–ecological systems frameworks caution policy makers that there are no panaceas for social–ecological challenges. They urge recognition of institutional diversity in the same ways others celebrate and defend biological diversity (Ostrom, 2005). What is more, despite the normative ecosystem services framework of the CBD, every major biodiversity status report calls for transformative change (e.g., Intergovernmental Science‐Policy Platform on Biodiversity and Ecosystem Services [IPBES], 2019). The importance of cultural (or biocultural) diversity and different ways of valuing and relating to the natural world has been belatedly recognized in conservation (IPBES, 2022). In addition, there is the perverse individuality of human beings, history, and questions of free will and counterfactuals to contend with (Pooley, 2018).

Incorporating the study of humans into conservation studies requires careful consideration of the language and concepts that conservation biology hardwired into the field. The languages of physics and systems science still carry authority in areas they have little business in. A decade ago, when I began thinking about human–wildlife coexistence, I was struck by Carter and Linnell's (2016) formulation of coexistence with predators as a resilient “state.” Colleagues and I adapted this to a formulation for coexistence generalized to all wildlife: “a sustainable though dynamic state, where humans and wildlife co‐adapt to sharing landscapes and human interactions with wildlife are effectively governed to ensure wildlife populations persist in socially legitimate ways that ensure tolerable risk levels” (Pooley et al., 2020).

This definition includes important statements on resilience and justice. And yet, peoples and societies are not, on reflection, usefully analyzed and understood through the language of physics or systems engineering. The behavior of human beings from different places, cultural backgrounds, histories, and societies cannot be studied like the behavior of particles or electrons to which universal laws apply. The same may be said for other animals. Particular communities of humans and wild animals interact in quite different ways (Pooley, 2024).

Human social and psychological complexities are not reducible to general laws, and yet the initiatives that ushered human dimensions into the conservation biology mainstream appear to offer this. The languages of resilience thinking and social–ecological systems thinking reach for universal frameworks, concepts, laws, and processes to explain (and govern) humans and their societies (e.g., Anderson et al., 2021; Resilience Alliance, 2024).

However, those who have followed Elinor Ostrom and others’ attempts to work out the rules of social–ecological systems and institutions for environmental governance will be familiar with the apparently unending proliferation of influential variables (Partelow, 2018). The popular theory of planned behavior seems also to generate layers upon layers of interacting shaping variables, once applied in sophisticated ways to specific situations (Jochum et al., 2014).

There seems to be an inevitable slide toward creating a map as big as the territory. This complexity cannot be wished away, but perhaps conservationists can consider how best to engage with it. What kinds of frameworks and engagements will be fruitful and which will lead only deeper into describing complexity and cataloguing decline more completely?

In the field of human‐wildlife interactions, the IUCN Human‐Wildlife Conflict and Coexistence Specialist Group (HWC SG) created a set of guidelines that direct conservationists towards the key elements and questions they should consider when approaching human‐wildlife conflicts (International Union for Conservation of Nature [IUCN], 2023). This is not a prescription for best practice, and rather than standard procedures, it offers 5 general principles.

However, if reducing negative human‐wildlife interactions is to be incorporated into the GBF, guidelines are not sufficient. Goals or targets included in the framework must have associated interventions with indicators to enable measurable outcomes. The HWC SG is now tasked with quantifying and reducing human‐wildlife conflict.

The approach has been to develop a 3‐part composite indicator that incorporates conflict incidence, social tolerance for wildlife (or not), and what approaches and resources are available and being implemented to reduce conflicts (IUCN, 2024a). Each indicator will have to be assessed by all CBD signatories and so will have to work across all countries. Speaking at CBD COP16 in Colombia, HWC SG Chair Alexandra Zimmermann noted: “[I]t is essential that we ensure adequate monitoring is in place [and offer] Parties a way to capture a number of aspects of human‐wildlife conflict across different national contexts, priorities and needs” (IUCN, 2024b).

Taking a step back from such daunting endeavors, it seems worthwhile for conservationists to reflect on where efforts will best be concentrated in the face of the biodiversity crisis. Current policy frameworks and conservation science have so far done too little to slow it. Biodiversity is crashing, and quite a lot is known about where and why. Conventions, policies, management actions, and regulations have so far not turned the tide.

Many of my students find the enumeration of biodiversity decline and explanations of causes—together with traditional approaches to defending dwindling wildlife populations and protected areas—profoundly reactive, conservative, and depressing. E.J. Milner‐Gulland's Conservation Optimism initiative is one prominent response to this problem (Conservation Optimism, 2024).

In the field of restoration ecology, rewilding has emerged as a popular movement, generating positive energy among its adherents (and fury among its detractors). It offers a positive vision of the future of humans living with wildlife on a fast‐transforming planet; it accommodates novelty and change and the agency of the wild (Rewilding Europe, 2024).

In the field of human–wildlife interactions, coexistence could be viewed similarly. The term has attained traction in conservation research and policies and in public discourse. However, some colleagues regard it as a fad, or at best, old wine in a new bottle adding a politically correct gloss to existing approaches (land sharing, tolerance, etc.). Others are energized by the possibilities of developing more inclusive conservation approaches that incorporate culture, identity, social justice, animal rights, and pathways to restorative justice. Some conservationists are working to develop interpretations of coexistence that can be integrated into conservation initiatives and thus go beyond a purely social movement buoyed by the zeitgeist (Marchini et al., 2023; Pooley et al., 2022).

However, the main contribution of coexistence thinking will not be to generate new universal rules to explain and govern how to live successfully with wildlife. This seems important to clarify as coexistence is being absorbed into the conservation mainstream. There is pressure to fix the definition of *human–wildlife coexistence* and develop standards for mitigating conflicts and fostering coexistence (Gross et al., 2021).

Coexistence could easily be subsumed into current frameworks by being reduced to a vague aspiration tacked onto existing approaches that prioritize conflict mitigation. In this framing, when the conflict mitigation work has solved all conflicts, then coexistence may emerge. Reducing coexistence to this would be to lose the essence of its difference.

What is coexistence then? In my view (one among a healthy diversity of views), coexistence is a social contract between consenting individuals representing all the social groups comprising a larger community of humans and wildlife sharing a landscape. Adherents willingly coadapt and live so that wildlife can persist within agreed boundaries of what the land and its nonhuman inhabitants require to flourish, and within what is acceptable, agreed, and necessary for the human communities to persist and flourish.

Although I am cautious about proposing metrics (the field is not quite there yet), the notable skepticism in conservation science and policy circles about the possibility of clearly identifying and assessing coexistence leads me to suggest some of what needs to be done. At base, in common with existing conservation approaches, it should be determined whether populations of wildlife are viable and sustainable. Methods must be developed to assess the status of human–nonhuman species interactions, including assessing the capacities of species and what they need to thrive, not merely exist (Pooley, 2022). There must be representative local institutions in place for responding to negative incidents and resolving conflicts with transparent and just decision‐making processes. Representatives of key stakeholder groups should be satisfied that the impacts and costs of living with wildlife are tolerable, and impacts should be collaboratively monitored with agreed metrics (Newing et al., 2024). Above all, there must be willingness to coexist and resources to enable it.

Fostering human–wildlife coexistence is about codeveloping a positive vision for what is wanted and recognizing and supporting coexistence where it already exists. It is about agreeing on what values and evidence should guide the processes for achieving this. A coexistence approach is less oriented to mitigating negative impacts (reducing body counts, offsetting losses) and correcting undesirable human behavior (to live in peace, it is insufficient to focus on preventing war) than traditional conservation approaches. Coexistence is not a substitute for existing methods for managing human–wildlife interactions. It offers something additional, something that is missing.

What is missing? Sometimes, conservation as a field seems to have, rather than a vision for a desirable future, instead an interim vision for the best ways to get there (better science informing better policy and management). The focus is on the rigors of preventing biodiversity loss, rather than on achieving clarity on the visions of what living well in nature might look like.

It is insufficient and uninspiring to say nature conservation's ultimate goal is to find the best ways to prevent decline. If transformational thinking is required—for example, asking what an economy is for (and for whom), rather than deducing economic laws and predicting their outcomes (Raworth, 2018)—then should not conservationists be asking what conservation is for (and for whom)? What is the vision for what conservationists want the world (or a region, or a landscape) to be transformed into, and who needs to be a part of that?

How should the coexistence of people and wildlife in functioning ecosystems (some of which locals agree to spare for wildlife) be pursued? Rather than standards for coexistence, founded on notions of universality and uniformity and top‐down governance through certification and assessment, coexistence may be best developed collaboratively by formulating shared visions and coexistence principles relevant to specific landscapes. Principles are propositions that serve as the foundation for a system of behavior. They influence how something should be done and for what purpose. They offer guidance, not blueprints.

Developing such visions requires agreeing on the spatial boundaries of the coexistence landscape. There must be consideration of the physical, ecological, economic, customary, spiritual, and legal dimensions of land use, governance, and ownership. Deliberations should also consider the historical and ongoing dynamics of human and animal land use.

There are no panaceas in conservation—the challenges are too complicated and diverse. However, acknowledging this diversity and complexity in nature and human societies requires neither giving in to anarchy nor trying to impose blueprints for saving the natural world. Instead, conservationist could try discovering and fostering visions for coexistence for communities of humans and wildlife in particular landscapes. To these visions, they can bring their knowledge, methods, tools, and aspirations, with generosity and humility. Coexistence does exist in diverse places and ways. Some of these may be transferable.
